# An Electrocatalytic/Heterogeneous Catalytic Cascade for Selective Production of Propylene Oxide via Anodic H_2_O_2_ Generation

**DOI:** 10.1002/anie.202521921

**Published:** 2025-12-30

**Authors:** Shubhadeep Chandra, Anirudha Shekhawat, Adarsh Koul, Ridha Zerdoumi, Lejing Li, Wolfgang Schuhmann

**Affiliations:** ^1^ Analytical Chemistry – Center for Electrochemical Sciences (CES) Faculty of Chemistry and Biochemistry, Ruhr University Bochum, Universitätsstraße 150 D‐44780 Bochum Germany; ^2^ Chair for Materials Discovery and Interfaces Institute for Materials Faculty of Mechanical Engineering, Ruhr University Bochum, Universitätsstraße 150 44801 Bochum Germany

**Keywords:** Anodic hydrogen peroxide, Cascade system, Gas diffusion layer, Propylene epoxidation, TS‐1 immobilization

## Abstract

Propylene oxide, a key intermediate with wide applications in the plastics industry, is still mainly produced by energy‐intensive and environmentally non‐sustainable processes. Electrochemically assisted epoxidation of propylene is emerging as a sustainable and atom‐efficient method for highly selective synthesis of propylene oxide. Here, we introduce a cascade strategy that combines anodic H_2_O_2_ generation with propylene epoxidation at a porous layer of immobilized titanium silicate (TS‐1). A ZnWO_4_ electrocatalyst was developed for efficient anodic H_2_O_2_ generation. To maximize local reactant concentrations, we designed an integrated TS‐1‐immobilized gas diffusion layer, which facilitates rapid propylene transport to the triple‐phase boundaries, while preventing TS‐1 loss and avoiding separation issues common in solution‐phase heterogeneous catalytic systems. Furthermore, an acetonitrile‐bicarbonate containing electrolyte system was optimized to facilitate direct utilization of H_2_O_2_ for propylene epoxidation, leading to 98% H_2_O_2_ utilization efficiency and over 97% selectivity for propylene oxide. This work offers a safer and greener alternative for propylene oxide production and broadens the application of electrochemically generated H_2_O_2_ from water oxidation for selective oxygenation reactions.

## Graphical abstract

This work demonstrates a cascade strategy for the selective synthesis of propylene oxide (PO) by coupling anodic H_2_O_2_ generation with TS‐1 catalyzed propylene epoxidation. By immobilizing TS‐1 on a gas diffusion layer, the system achieves high PO productivity with efficient H_2_O_2_ utilization, providing a safer and greener alternative to conventional PO production.

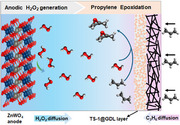

## Introduction

Propylene oxide (PO) is a crucial intermediate in producing various high‐value chemicals such as polyester, polyurethane, and propylene glycol.^[^ ^1^, ^2^, ^3^ ^]^ Therefore, developing efficient and environmentally friendly methods for its selective synthesis is highly important. The traditional chlorohydrin process produces significant chemical waste and wastewater, raising serious environmental concerns.^[^ ^4^, ^5^ ^]^ As a result, direct oxidation of propylene using molecular oxygen (O_2_) has been extensively studied as a greener alternative.^[^ ^2^, ^3^, ^6^ ^]^ However, this method faces challenges due to the low selectivity for PO, mainly because of allylic hydrogen abstraction and overoxidation to CO_2_.^[^ ^7^ ^]^ To overcome these limitations, researchers have focused on alternative sustainable approaches, particularly the use of titanium silicate (TS‐1) as catalyst combined with hydrogen peroxide (H_2_O_2_) as an oxidant for the selective epoxidation of propylene.^[^ ^8^, ^9^ ^]^ However, the mainstream anthraquinone process for H_2_O_2_ production involves sequential hydrogenation and oxidation steps of anthraquinone and H_2_O_2_ extraction, which hinder seamless integration with subsequent epoxidation.^[^ ^10^ ^]^ Additionally, transportation and storage of highly concentrated H_2_O_2_ increase both costs and safety risks. These limitations have prompted researchers to seek more promising alternatives that integrate in situ generated H_2_O_2_ with epoxidation to intensify the cascade reactions toward value‐added feedstocks.

**Scheme 1:**
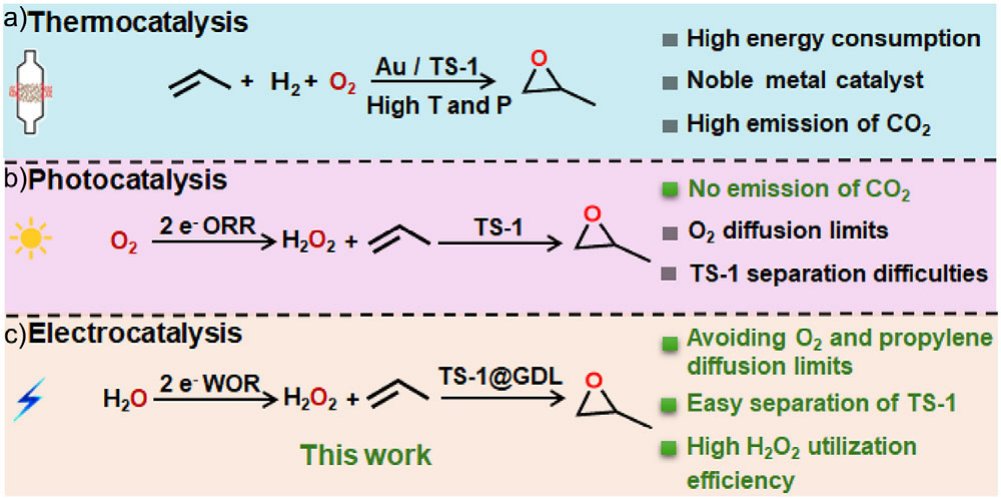
Schematic comparison of PO production pathways using in situ generated H_2_O_2_.

Haruta et al. demonstrated a strategy to generate PO over Au‐supported catalysts in conjunction with TS‐1, employing in situ generated H_2_O_2_ from H_2_ and O_2_ (Scheme 1a).^[^ ^11^ ^]^ While this approach eliminates the need for commercial H_2_O_2_, it still relies on noble metal catalysts, explosive hydrogen gas, and high reaction temperatures, which pose safety and cost challenges. ^[^ ^12^, ^13^ ^]^ Electrocatalytic synthesis has recently emerged as an attractive strategy for producing PO.^[^ ^14^, ^15^, ^16^ ^]^ Kwak and colleagues introduced a photo‐electro‐heterogeneous catalytic system for the epoxidation of propylene to PO, utilizing in situ generated H_2_O_2_ from O_2_ via the oxygen reduction reaction (ORR) (Scheme 1b).^[^ ^17^ ^]^ TS‐1 suspended in the catholyte solution was used to facilitate the epoxidation of propylene with the in situ generated H_2_O_2_. However, the kinetics of the electrocatalytic or photocatalytic ORR are limited by the poor solubility and low diffusion coefficient of O_2_ in aqueous media.^[^ ^18^ ^]^ Additionally, the ORR requires a continuous supply of O_2_ or air to the cathode surface, increasing operational costs and complexity.^[^ ^19^ ^]^ In this context, the anodic generation of H_2_O_2_ by H_2_O oxidation offers a sustainable and modular alternative for in situ H_2_O_2_ generation without O_2_ feed.^[^ ^19^, ^20^ ^]^ Additionally, when TS‐1 is suspended in the electrolyte, catalyst agglomeration and recovery, low solubility and poor mass transfer of propylene can dramatically reduce overall epoxidation efficiency.^[^ ^21^, ^22^ ^]^ Therefore, an argument could be made that direct epoxidation of propylene on a gas diffusion layer (GDL) incorporated with TS‐1 can significantly simplify the process. The porous structures of GDLs can facilitate rapid mass transfer of propylene molecules, enabling direct interaction with TS‐1 and anodically generated H_2_O_2_ at the triple‐phase boundary. We hypothesize that such integration overcomes limitations of heterogeneous catalyst suspensions and mitigates issues related to catalyst loss and separation costs, providing a continuous, modular, and environmentally friendly route for selective PO synthesis under mild conditions.

**Scheme 2:**
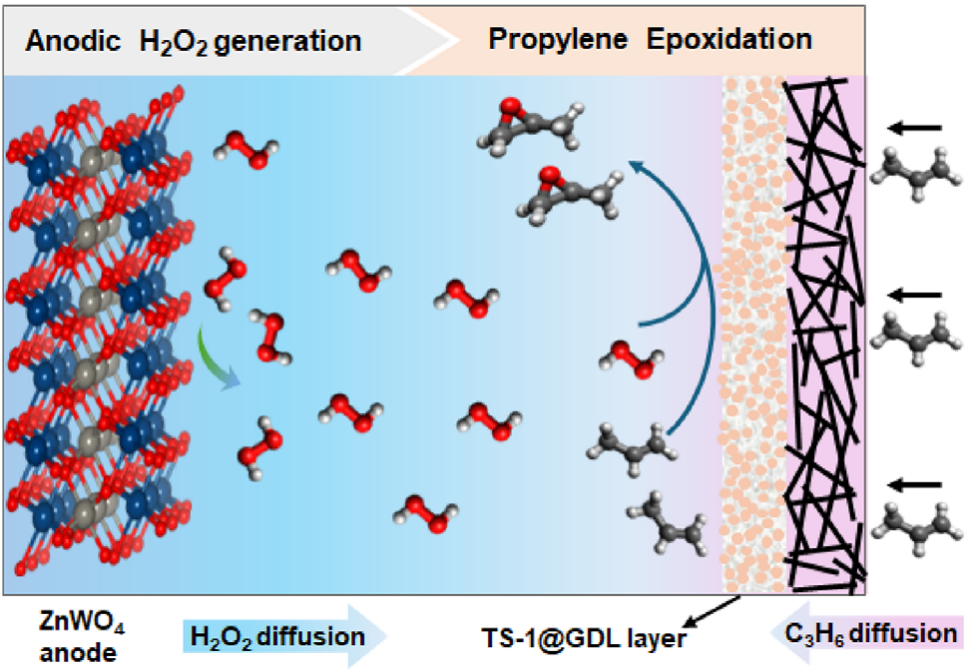
Scheme of PO production by coupling anodic in situ H_2_O_2_ generation with epoxidation of propylene over a TS‐1 modified GDL.

To approach this vision, we demonstrate a proof‐of‐concept system that couples anodic H_2_O_2_ generation with propylene epoxidation over self‐assembled TS‐1 incorporated into a GDL (TS‐1@GDL) in a model flow‐through electrolyzer (Scheme 2). A novel ZnWO_4_ catalyst was developed, achieving high Faradaic efficiency (FE) for H_2_O_2_ (FE_H2O2_) in 2 M KHCO_3_/K_2_CO_3_ as electrolyte while maintaining excellent durability. The ZnWO_4_ anode is strategically positioned adjacent to the TS‐1@GDL, allowing freshly generated H_2_O_2_ from the anode to diffuse directly to the triple phase boundaries with minimal decomposition, thereby maximizing the H_2_O_2_ utilization efficiency for propylene epoxidation. Moreover, a compatible acetonitrile/KHCO_3_/K_2_CO_3_ solvent system was optimized to directly utilize electrochemically generated H_2_O_2_ for the epoxidation of propylene. As a result, this integrated system achieves a PO production rate of 62 µmol cm^−2^ h^−1^, with PO selectivity exceeding 97%.

## Results and Discussion

### Anode Material Characterizations

**Figure 1:**
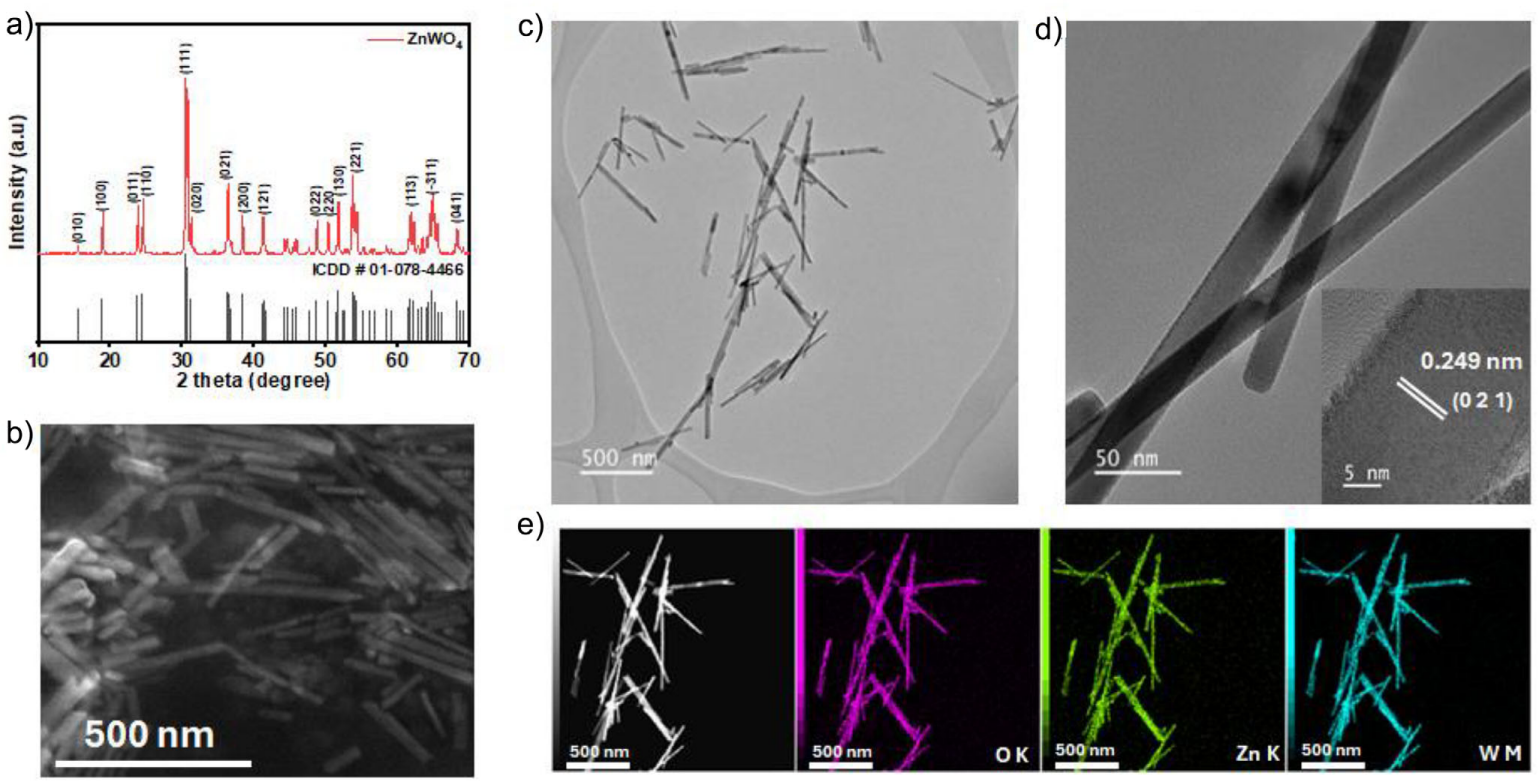
Characterizations of ZnWO_4_ catalyst. a) XRD pattern of ZnWO_4_ nanorods. b) SEM image of ZnWO_4_ nanorods. c) TEM image of ZnWO_4_ nanorods. d) High‐resolution TEM (HR‐TEM) image of ZnWO_4_ nanorods. e) STEM images with corresponding EDS elemental mapping of ZnWO_4_ nanorods.

To perform propylene epoxidation, ZnWO_4_ nanorods were employed as anode material to facilitate H_2_O_2_ generation. To increase the selectivity of H_2_O_2_, the anode should show weak adsorption to oxygen intermediates (*O, *OH, *OOH), thereby disfavoring the oxygen evolution reaction. ZnWO_4_ was chosen as the anode for anodic H_2_O_2_ production because of the closed‐shell configurations of Zn^2^⁺ and W^6^⁺. The ZnWO_4_ nanorods were synthesized via a solvothermal method, adapted from a previously reported procedure.^[^ ^23^ ^]^ ZnWO_4_ crystallizes in a monoclinic wolframite‐type structure, where both Zn^2+^ and W^6+^ occupy octahedral sites forming zig‐zag chains along the c‐axis.^[^ ^24^ ^]^ Distortion of ZnO_6_ and WO_6_ octahedra introduces unsaturated surface sites, which are favorable for facilitating catalytic reactions. The X‐ray diffraction (XRD) pattern of the as‐prepared sample matches well with the reference pattern of monoclinic ZnWO_4_ (ICDD # 01–078–4466, Space group: P2/c (13)), indicating the single‐phase nature of the sample.^[^ ^24^ ^]^ The sharp and well‐defined reflections at 15.5°, 18.9°, 23.8°, 24.6°, 30.5°, and 36.4° can be indexed to (010), (100), (011), (110), (111), and (021) crystal planes, respectively, indicating high crystallinity of the as‐prepared material (Figure 1a). Scanning electron microscopy (SEM) image in Figure 1b reveals that the ZnWO_4_ catalyst consists of well‐defined, straight, and elongated nanorods, which is confirmed by transmission electron microscopy (TEM) images (Figure 1c). High‐resolution TEM (HR‐TEM) analysis (Figure 1d), taken from the highlighted region, shows distinct lattice fringes with an interplanar spacing of 0.249 nm, corresponding to the (021) plane of ZnWO_4_ (inset of Figure 1d). Elemental mapping using scanning transmission electron microscopy (STEM‐EDS) confirms a uniform distribution of Zn, W, and O (Figure 1e). X‐ray photoelectron spectroscopy (XPS) was employed to analyze the valence states and the composition of the near‐surface region of the freshly prepared ZnWO_4_ anode (Figure S1). The deconvolution of the high‐resolution W 4*f* spectra indicates the presence of mixed W^4+^, W^5+^ and W^6+^ valence states. The near‐surface composition was found to be 77.1% ± 0.1, 9.0% ± 0.1, and 13.9% ± 0.1 for O, Zn, and W, respectively (Figure S2).

### Anodic H_2_O_2_ Generation

The ZnWO_4_ anode was specifically designed for selective H_2_O_2_ production (see Supporting Information) and exhibits a uniform distribution of individually dispersed particles on the electrode surface, as confirmed by morphological analysis (Figure S3). Previous studies have demonstrated that employing a HCO_3_^−^/CO_3_^2−^ buffer system as anolyte can substantially enhance the selectivity of anodic H_2_O_2_ formation.^[^ ^25^, ^26^ ^]^ The strong buffering capacity mitigates local pH shifts during electrolysis, which is essential for sustaining a high interfacial HCO_3_^−^ concentration. Within this equilibrium, HCO_3_^−^ ions play a critical role in mediating anodic H_2_O_2_ production. CO_3_^2−^ ions and protons released from the anode continuously regenerate HCO_3_^−^, ensuring a high HCO_3_^−^ concentration at the anode/electrolyte interface.^[^ ^19^, ^25^ ^]^ The H_2_O_2_ generation performance was evaluated based on the accumulated H_2_O_2_ concentration in the anolyte over time.

**Figure 2:**
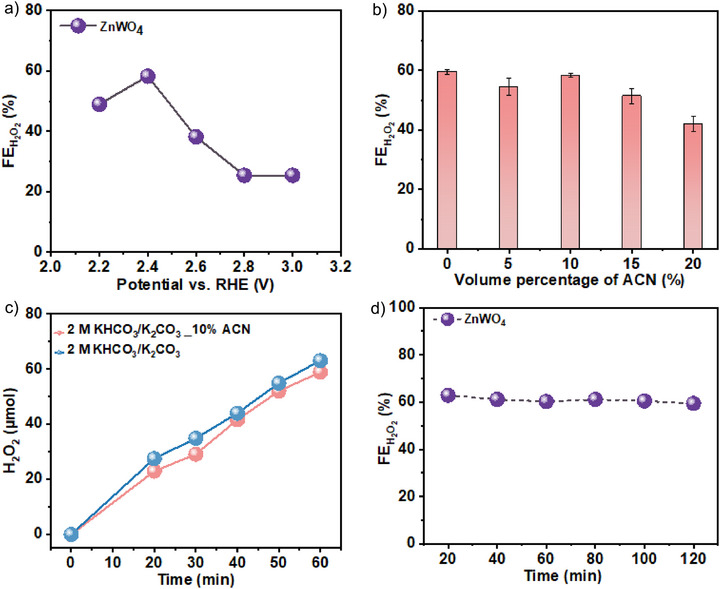
H_2_O_2_ generation performance of ZnWO_4_ anode. a) FE_H__2__O__2_ of the prepared ZnWO_4_ anode as a function of anode potential in 2 M KHCO_3_/K_2_CO_3_ electrolyte (pH 9). b) FE_H__2__O__2_ of the ZnWO_4_ anode at 2.4 V versus RHE in 2 M KHCO_3_/K_2_CO_3_ electrolyte (pH 9) mixed with different volume percentages of ACN. Error bars represent the standard deviation from three independent experiments. c) H_2_O_2_ generation with time at 2.4 V versus RHE on the ZnWO_4_ anode in 2 M KHCO_3_/K_2_CO_3_ mixed with 10% ACN and without ACN. d) Repeated H_2_O_2_ performance tests on ZnWO_4_ anode at 2.4 V versus RHE over 120 min in 2 M KHCO_3_/K_2_CO_3_ electrolyte (pH 9) containing 10% ACN. FE_H__2__O__2_ calculated after every 20 min, with the electrolyte refreshed each cycle.

Linear sweep voltammetry (LSV) was performed to assess the electrocatalytic performance of ZnWO_4_ anode in 2 M KHCO_3_ and a mixture of KHCO_3_/K_2_CO_3_ at pH 9 (Figure S4). In the KHCO_3_/K_2_CO_3_ electrolyte (pH 9), the ZnWO_4_ anode exhibits a higher current density compared to pure KHCO_3_ solution. The FE for H_2_O_2_ formation (FE_H__2__O__2_) was measured across a potential range of 2.2 to 3.0 V versus RHE (Figure 2a). In 2 M KHCO_3_, the ZnWO_4_ anode achieved a maximum FE_H__2__O__2_ of 45% at an applied potential of 2.4 V versus RHE (Figure S5). In a mixture of KHCO_3_/K_2_CO_3_ with a pH of 9, the ZnWO_4_ anode exhibits a higher FE_H__2__O__2_, reaching a maximum FE_H__2__O__2_ of 60% at 2.4 V versus RHE (overpotential ≈ 640 mV), demonstrating high selectivity for H_2_O_2_ production (Figure 2a). At potentials above 2.4 V, the FE_H__2__O__2_ declines due to the competing oxygen evolution reaction (OER). Switching to a pure K_2_CO_3_ electrolyte did not lead to a further increase in FE_H__2__O__2_ (Figure S6). These results demonstrate that the mixture of KHCO_3_/K_2_CO_3_ electrolytes significantly enhances H_2_O_2_ production at the ZnWO_4_ anode compared to pure KHCO_3_ or K_2_CO_3_ electrolytes.

PO is prone to hydrolysis under alkaline conditions, resulting in ring‐opening and formation of propylene glycol.^[^ ^27^ ^]^ To mitigate this, a mixed solvent system comprising acetonitrile (ACN) and an aqueous KHCO_3_/K_2_CO_3_ electrolyte was employed.^[^ ^28^ ^]^ However, we were not sure if the introduction of ACN into the KHCO_3_/K_2_CO_3_ mixture affects H_2_O_2_ generation performance and the stability of the ZnWO_4_ anode. To elucidate these effects, we systematically investigated solvent compositions by varying the volume percentages of ACN in aqueous‐organic mixtures for H_2_O_2_ generation. The FEH_2_O_2_ remains relatively stable across the ACN volume percentages ranging from 5% to 10% in KHCO_3_/K_2_CO_3_ electrolyte (pH 9) at 2.4 V versus RHE (Figure 2b). The ZnWO_4_ anode exhibits a FEH_2_O_2_ of approximately 60% in the presence of 10% ACN, comparable to that observed in aqueous KHCO_3_/K_2_CO_3_ electrolyte, indicating that its catalytic activity and H_2_O_2_ production efficiency are preserved in 10% ACN aqueous‐organic medium (Figure 2b).

At higher ACN contents, the FEH_2_O_2_ declines, likely due to phase separation and increased turbidity, which impair mass transport of HCO_3_^−^ anions and electron transfer processes. The H_2_O_2_ generation rate was also measured at different volume percentages of ACN in 2 M KHCO_3_/K_2_CO_3_ electrolyte. The ZnWO_4_ anode achieves a H_2_O_2_ generation rate exceeding 60 µmol cm^−2^ h^−1^ at 2.4 V versus RHE in 2 M KHCO_3_/K_2_CO_3_ electrolyte containing 10% ACN, similar to that without ACN (Figure S7). The H_2_O_2_ concentration increases nearly linearly over 60 min, confirming stable and efficient production with minimal H_2_O_2_ decomposition (Figure 2c). Durability was assessed by repeating H_2_O_2_ electrosynthesis at 2.4 V versus RHE (Figure 2d) in 2 M KHCO_3_/K_2_CO_3_ electrolyte containing 10% ACN. To minimize changes in both the electrolyte composition and pH, the electrolyte was refreshed every 20 min. The FE_H__2__O__2_ was measured for each test cycle, and a FE_H__2__O__2_ of 60% was maintained after 120 min of continuous electrolysis. The LSV recorded after 120 min stability test shows no apparent decrease in current density (Figure S8). XRD patterns of ZnWO_4_ before and after electrolysis show no noticeable changes, confirming its structural integrity during the electrolysis (Figure S9). The nanorod‐like morphology of the ZnWO_4_ catalyst on the anode surface was retained after the stability test in the aqueous‐organic medium (Figure S10). The comparison of high‐resolution XPS spectra of Zn 3*p* and W 4*f* before and after the stability test reveals no significant changes in the valence states of the metal ions (Figure S1). Moreover, the near‐surface composition of the catalysts remains unchanged after electrolysis (Figure S2). The stability of ZnWO_4_ under harsh oxidation conditions was further evaluated by quantifying the concentration of leached metals in the anolyte. Inductively coupled plasma mass spectrometry (ICP‐MS) analysis showed that after 120 min of electrolysis at 2.4 V versus RHE, only trace amounts of Zn and W were detected in the anolyte, confirming the high stability of the ZnWO_4_ anode at operational conditions. The nearly unchanged LSV before and after the stability test, along with the preserved catalyst morphology after electrolysis, demonstrate the excellent durability of the ZnWO_4_ anode under highly oxidative conditions in 2 M KHCO_3_/K_2_CO_3_ electrolyte containing 10% ACN.

### Propylene Epoxidation with the Integrated Catalytic System

**Figure 3:**
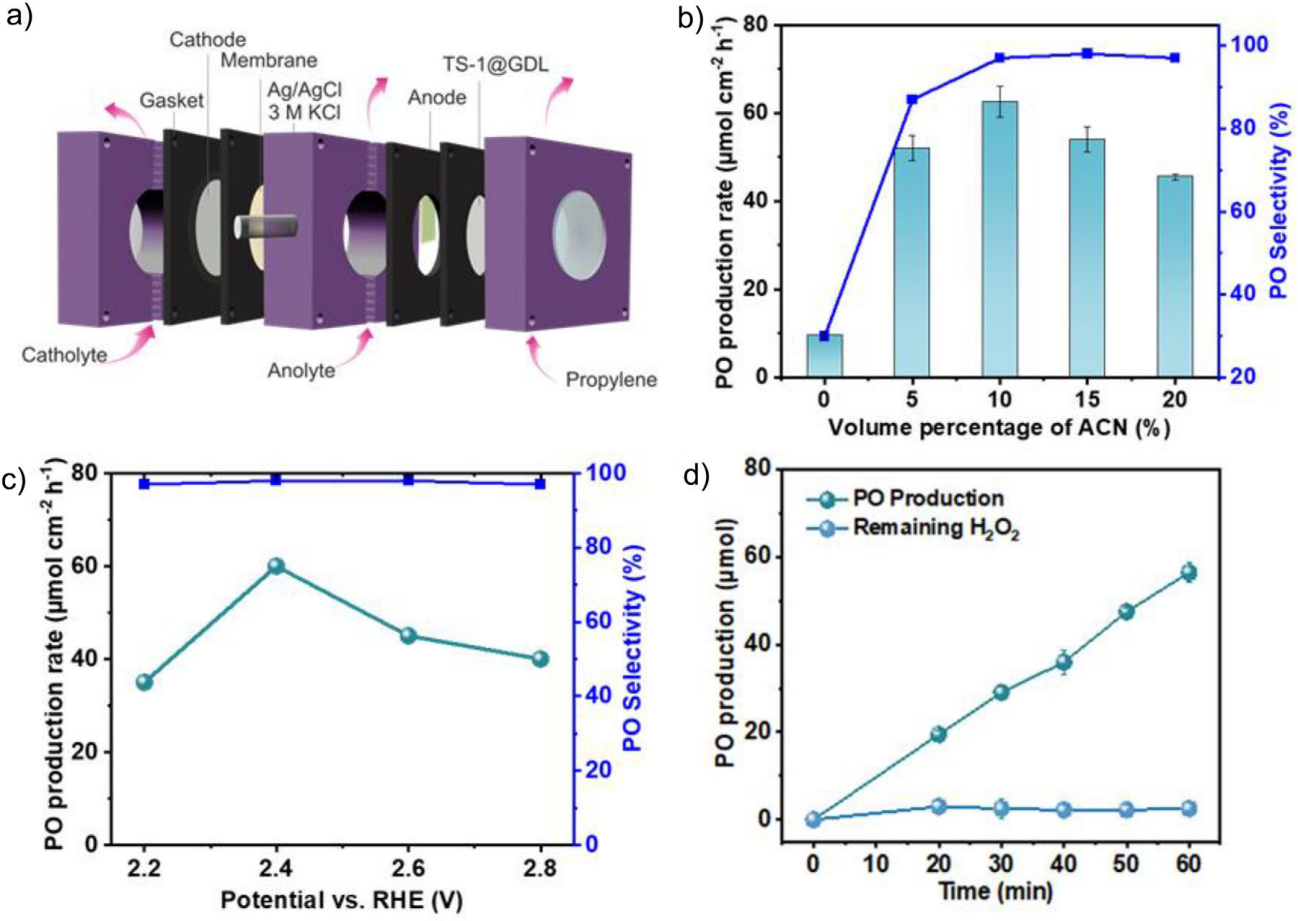
a) Schematic diagram of propylene epoxidation in a model flow‐through electrolyzer. b) Production rate and selectivity of PO at 2.4 V versus RHE in 2 M KHCO_3_/K_2_CO_3_ (pH 9) electrolyte mixed with different volume percentages of ACN. c) Production rate and selectivity of PO at different applied potentials in 2 M KHCO_3_/K_2_CO_3_ (pH 9) electrolyte containing 10% ACN. d) PO production and remaining H_2_O_2_ with time in an integrated electrocatalytic‐heterogeneous catalytic system in 2 M KHCO_3_/K_2_CO_3_ electrolyte (pH 9) containing 10% ACN. Error bars represent the standard deviation from three independent experiments.

To facilitate the epoxidation of propylene, TS‐1 was immobilized onto the GDL (TS‐1@GDL). Carbon nanotubes decorated Ni foam (CNTs@NF) served as the porous support of the GDL, providing a hierarchical porous architecture for an effective loading with TS‐1 particles (Figure S11).^[^ ^29^, ^30^ ^]^ TS‐1 remains securely anchored at the GDL surface, minimizing catalyst loss and aggregation typically observed in suspensions. Both anodic H_2_O_2_ generation and propylene epoxidation were investigated in a model flow‐through electrolyzer (Figure 3a, Figure S12). As illustrated in Figure 3a, the ZnWO_4_ anode was strategically positioned adjacent to TS‐1@GDL. This spatial configuration ensures the local high H_2_O_2_ concentration at the TS‐1 active sites, thereby improving both epoxidation efficiency and H_2_O_2_ utilization. The formation of PO was confirmed by ^1^H nuclear magnetic resonance (NMR) spectra which matched with a commercial PO standard (Figure S13) Propylene glycol (PG) was obtained as a major product when the reaction was performed in KHCO_3_/K_2_CO_3_ solution (pH 9) without ACN, due to the hydrolysis of PO, resulting in ring opening and formation of PG (Figure S14). Therefore, considering the stability of PO in KHCO_3_/K_2_CO_3_ solution, we sought to investigate the impact of the ACN volume percentage on the formation of PO. The formation of PG was immediately inhibited upon adding 5% ACN (Figures 3b and S14). The PO production rate increases with increasing ACN volume percentage, reaching a maximum of 62 µmol h^−1^ with 10% ACN in 2 M KHCO_3_/K_2_CO_3_ electrolyte (pH 9) with a PO selectivity exceeding 97%. The addition of ACN effectively suppresses PO hydrolysis and enhances the solubility of propylene gas in the reaction medium, increasing its availability for efficient epoxidation. This aqueous‐organic mixture provides a favorable environment for maintaining epoxide integrity and enables the direct use of electrochemically generated H_2_O_2_ from the ZnWO_4_ anode, improving overall selectivity and reaction efficiency. However, increasing the ACN fraction to 20% decreases the PO production rate, despite selectivity exceeding 98%, due to a decreased H_2_O_2_ generation (Figure 3b).

To explore the potential dependence of propylene epoxidation, chronoamperometry measurements were performed at different applied potentials in 2 M KHCO_3_/K_2_CO_3_ electrolyte (pH 9) containing 10% ACN. PO formation is negligible below 2.2 V versus RHE. Increasing the potential from 2.2 V to 2.4 V leads to a significant rise in PO production from 35 to 62 µmol h^−1^, following the trend of electrochemically generated H_2_O_2_ (Figure 3c). This indicates a strong correlation between H_2_O_2_ availability and epoxidation efficiency in the optimized 10% ACN electrolyte, suggesting that the H_2_O_2_ production rate is the most important factor determining the overall performance of PO production.

**Figure 4:**
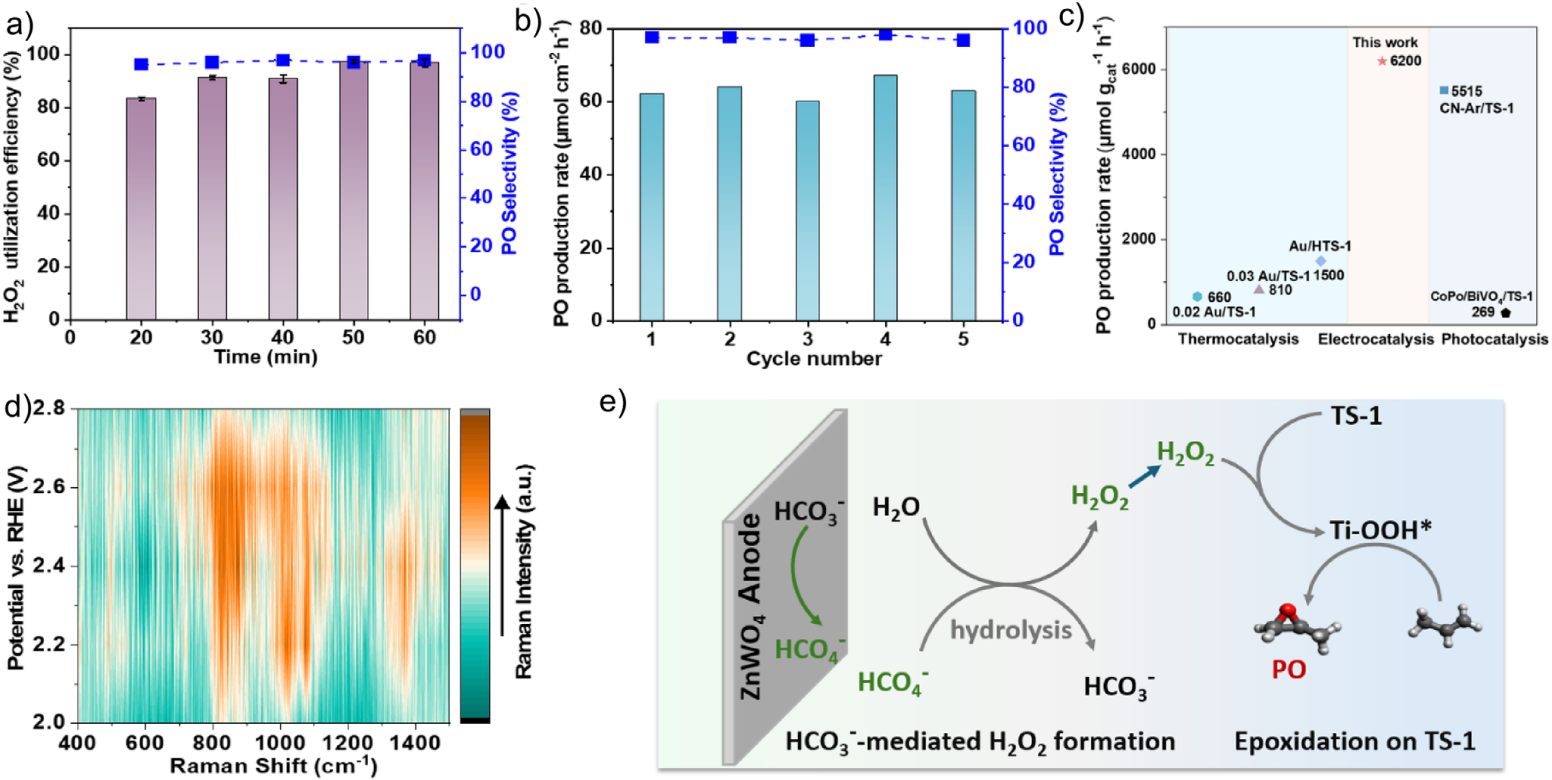
a) H_2_O_2_ utilization efficiency for production of PO over 60 min of electrolysis at 2.4 V versus RHE in 2 M KHCO_3_/K_2_CO_3_ electrolyte (pH 9) containing 10% ACN. b) Cyclic stability test of ZnWO_4_ anode and TS‐1@GDL for propylene epoxidation. c) Comparison of PO production rate over our system and previously reported catalysts using in situ generated H_2_O_2_. d) Potential‐dependent Raman spectra at immobilized TS‐1 during propylene epoxidation. e) The proposed reaction mechanism for PO formation via anodic H_2_O_2_ generation using immobilized TS‐1 in 2 M KHCO_3_/K_2_CO_3_ electrolyte containing 10% ACN. Error bars represent the standard deviation from three independent experiments.

To further enhance the catalytic performance, the amount of TS‐1 loaded on the GDL was optimized. The PO production rate increases with an increase in TS‐1 loading, implying the importance of efficient activation of H_2_O_2_ by TS‐1. The highest PO production rate was observed at a TS‐1 loading of 6 mg cm^−2^ (Figure S15). In the absence of the TS‐1 catalyst, neither PO nor PG was detected in the electrolyte solution, confirming that the TS‐1 catalyst is essential for propylene oxidation in the presence of in situ generated H_2_O_2_ (Figure S16). This also indicates that direct oxidation of propylene does not occur on the ZnWO_4_ anode surface during the electrolysis. To explore the effect of propylene flow rate on PO production, chronoamperometry measurements were performed at different propylene flow rates. The PO production rate showed only a slight increase with varying propylene flow rates, indicating that the flow rate has a minimal effect on PO production in our system (Figure S17). The H_2_O_2_ production rate is the limiting factor that determines the overall PO production. The production rate is a key parameter for evaluating overall efficiency and scalability of the process. Under optimal conditions, PO concentrations increased almost linearly for 60 min, reaching a total of 62 µmol with a selectivity exceeding 97% (Figure S18). After 20 min, the H_2_O_2_ concentration remained steady, indicating that a steady‐state equilibrium was reached between the H_2_O_2_ production and the consumption for propylene epoxidation (Figure 3d). Initially, H_2_O_2_ utilization efficiency was relatively low, likely due to PO adsorption on TS‐1. However, efficiency improved over time, and after 40 min, the combined amount of PO and PG produced and residual H_2_O_2_ closely matched the total H_2_O_2_ generated in the absence of TS‐1, corresponding to a H_2_O_2_ utilization efficiency of 98% (Figure 4a). The cascade system achieves a maximum electron efficiency (EE) of 58% for PO and PG production at 2.4 V versus RHE, as shown in Figure S19 (details of the calculation are provided in the Supporting Information). Compared to a suspension of TS‐1 particles in the electrolyte, immobilizing TS‐1 on the GDL surface ensures sufficient exposure of active sites and facilitates more effective contact between propylene and TS‐1, leading to improved catalytic performance and higher PO production (Figure S20). Additionally, ACN also promotes PO production by solvent‐mediated effects that preserve the integrity of active species on TS‐1, compared to pure KHCO_3_/K_2_CO_3_ solution.^[^ ^31^, ^32^ ^]^

Next, we performed multiple catalytic runs using the same ZnWO_4_ electrode and TS‐1@GDL to evaluate long‐term durability. The electrolyte was replaced after each cycle. As shown in Figure 4b, both selectivity and PO production rate remain essentially unchanged at 97% and around 62 µmol cm^−2^ h^−1^, respectively. A decline in the PO production rate was observed after multiple uses of TS‐1@GDL, probably due to partial blockage of Ti active sites by organic residues. Notably, the catalytic performance was largely restored after regeneration by calcination (Figure S21). Furthermore, no distinct difference was observed in the SEM images of TS‐1@GDL after calcination compared to the fresh TS‐1@GDL (Figure S22). This indicates that the TS‐1 catalysts remained well anchored to the GDL with minimal detachment. These findings underscore the robustness and applicability of the integrated anodic H_2_O_2_ generation and propylene epoxidation system. The PO productivity achieved in this study exceeds that reported in previous reports for PO synthesis using in situ generated H_2_O_2_ (Figure 4c; Tables S1 and S2).^[^ ^17^, ^33^, ^34^, ^35^, ^36^ ^]^ Our integrated system provides a significant advancement by combining efficient in situ H_2_O_2_ generation and rapid PO formation under mild conditions.

To elucidate the potential reaction intermediates and active species produced during the reaction, we employed in situ electrochemical FTIR spectroscopy (Figure S23). In bicarbonate solutions, the formation of H_2_O_2_ is largely attributed to the oxidation of HCO_3_^−^ to percarbonate (HCO_4_^−^).^[^ ^19^, ^37^ ^]^ When the potential exceeds 2.0 V versus RHE, a new band appeared at 1297 cm^−1^, which cannot be assigned to carbonate and bicarbonate species (Figure S24). This transient feature is tentatively attributed to HCO_4_^−^, formed at the highly oxidative conditions, as previously reported.^[^ ^38^ ^]^ Local acidification resulting from proton release at the anode surface accelerates the hydrolysis of HCO_4_^−^, leading to the rapid formation and release of H_2_O_2_. This dynamic behavior renders the isolation and full characterization of the intermediate particularly challenging. We also employed operando Raman spectroscopy to detect the intermediates formed on the TS‐1 surface during electrolysis (Figure 4d). When the potential exceeds 2.0 V versus RHE, the characteristic band of TS‐1 at 829 cm^−1^, attributed to the key intermediate Ti─OOH*, can be observed.^[^ ^39^, ^40^, ^41^ ^]^ The formation of the Ti─OOH* intermediate on TS‐1 is closely associated with H_2_O_2_ production at the anode. Considering the oxidizing power of HCO_4_^−^, in principle, propylene could also be directly oxidized by in situ generated HCO_4_^−^ without TS‐1.^[^ ^42^ ^]^ However, control experiments using only commercial H_2_O_2_ in HCO_3_^−^ solution (under conditions known to form HCO_4_^−^) show no propylene conversion (Figure S25). This is likely due to the weak interaction between propylene and the aqueous‐phase oxidant HCO_4_^−^ and the rapid hydrolysis of HCO_4_^−^. Based on the results, a plausible reaction mechanism for the anodic H_2_O_2_ generation coupled tandem propylene epoxidation system is proposed (Figure 4e). HCO_4_^−^ formed on the ZnWO_4_ anode surface rapidly hydrolyzes to H_2_O_2_, which is subsequently activated by TS‐1 to form the reactive Ti─OOH* intermediate. This Ti─OOH* intermediate selectively oxidizes propylene to PO, highlighting the essential role of TS‐1 in converting the in situ generated H_2_O_2_ into the active TiOOH* species and thereby driving the cascade epoxidation reaction.

## Conclusion

We have developed a sustainable electrocatalytic/heterogeneous catalytic cascade for the selective synthesis of propylene oxide (PO) by coupling anodic in situ H_2_O_2_ generation with immobilized TS‐1 catalyzed propylene epoxidation. A gas diffusion layer (GDL) incorporating immobilized TS‐1 nanoparticles enables efficient and selective epoxidation of propylene under mild conditions while avoiding issues typically observed in TS‐1 suspension, including catalyst loss, TS‐1 separation. The developed ZnWO_4_ anode showed high selectivity for H_2_O_2_ production, achieving a FE of 60% with excellent durability in 2 M KHCO_3_/K_2_CO_3_ electrolyte containing 10% acetonitrile. Using this ZnWO_4_ anode in combination with immobilized TS‐1@GDL, the integrated system exhibited excellent performance for PO synthesis, achieving a high production rate of 62 µmol cm^−2^ h^−1^, a PO selectivity exceeding 97% and a H_2_O_2_ utilization efficiency of 98%. The strategic positioning of the ZnWO_4_ anode adjacent to the TS‐1@GDL enables freshly generated H_2_O_2_ to diffuse directly to the triple phase boundaries with minimal decomposition, thereby maximizing H_2_O_2_ utilization and significantly enhancing PO production rate. This work demonstrates the first application of anodically generated H_2_O_2_ for propylene epoxidation at the triple phase boundaries, representing a significant advance over existing methods of PO production. More broadly, this study establishes a generalizable cascade strategy for harnessing anodically generated H_2_O_2_, highlighting a key step toward integrating electrochemical techniques into industrially relevant oxidation processes.

## Conflict of Interests

The authors declare no conflict of interest.

## Supporting information



Supporting Information

## Data Availability

The data that support the findings of this study are available from the corresponding author upon reasonable request.

## References

[1] J. Herzberger , K. Niederer , H. Pohlit , J. Seiwert , M. Worm , F. R. Wurm , H. Frey , Chem. Rev. 2016, 116, 2170–2243, 10.1021/acs.chemrev.5b00441. 26713458

[2] Y. Lei , F. Mehmood , S. Lee , J. Greeley , B. Lee , S. Seifert , R. E. Winans , J. W. Elam , R. J. Meyer , P. C. Redfern , D. Teschner , R. Schlögl , M. J. Pellin , L. A. Curtiss , S. Vajda , Science 2010, 328, 224–228, 10.1126/science.1185200. 20378815

[3] S. Ghosh , S. S. Acharyya , R. Tiwari , B. Sarkar , R. K. Singha , C. Pendem , T. Sasaki , R. Bal , ACS Catal. 2014, 4, 2169–2174, 10.1021/cs5004454.

[4] J. Teržan , M. Huš , B. Likozar , P. Djinović , ACS Catal. 2020, 10, 13415.

[5] W. R. Leow , Y. Lum , A. Ozden , Y. Wang , D.‐H. Nam , B. Chen , J. Wicks , T.‐T. Zhuang , F. Li , D. Sinton , E. H. Sargent , Science 2020, 368, 1228–1233, 10.1126/science.aaz8459. 32527828

[6] F. Cavani , J. H. Teles , ChemSusChem. 2009, 2, 508–534, 10.1002/cssc.200900020. 19536755

[7] S. J. Khatib , S. T. Oyama , Catal. Rev. Sci. Eng 2015, 57, 306–344, 10.1080/01614940.2015.1041849.

[8] C. P. Gordon , H. Engler , A. S. Tragl , M. Plodinec , T. Lunkenbein , A. Berkessel , J. H. Teles , A.‐N. Parvulescu , C. Copéret , Nature 2020, 586, 708–713, 10.1038/s41586-020-2826-3. 33116285

[9] W. Li , L. Chen , M. Qiu , W. Li , Y. Zhang , Y. Zhu , J. Li , X. Chen , ACS Catal. 2023, 13, 10487–10499, 10.1021/acscatal.3c02206.

[10] J. M. Campos‐Martin , G. Blanco‐Brieva , J. L. G. Fierro , Angew. Chem. Int. Ed. 2006, 45, 6962–6984, 10.1002/anie.200503779. 17039551

[11] T. Hayashi , K. Tanaka , M. Haruta , J. Catal. 1998, 178, 566.

[12] A. K. Sinha , S. Seelan , S. Tsubota , M. Haruta , Angew. Chem. Int. Ed. 2004, 43, 1546–1548, 10.1002/anie.200352900. 15022229

[13] B. S. Uphade , T. Akita , T. Nakamura , M. Haruta , J. Catal. 2002, 209, 331.

[14] M. Chung , J. H. Maalouf , J. S. Adams , C. Jiang , Y. Román‐Leshkov , K. Manthiram , Science 2024, 383, 49–55, 10.1126/science.adh4355. 38175873

[15] M. Chi , J. Ke , Y. Liu , M. Wei , H. Li , J. Zhao , Y. Zhou , Z. Gu , Z. Geng , J. Zeng , Nat. Commun. 2024, 15, 3646, 10.1038/s41467-024-48070-1. 38684683 PMC11059342

[16] S. Chandra , A. Koul , J. Zhang , S. Seisel , W. Schuhmann , Chem. – Eur. J. 2024, 30, e202303830, 10.1002/chem.202303830. 38271542

[17] M. Ko , Y. Kim , J. Woo , B. Lee , R. Mehrotra , P. Sharma , J. Kim , S. W. Hwang , H. Y. Jeong , H. Lim , S. H. Joo , J.‐W. Jang , J. H. Kwak , Nat. Catal. 2022, 5, 37–44, 10.1038/s41929-021-00724-9.

[18] Q. Zhang , M. Zhou , G. Ren , Y. Li , Y. Li , X. Du , Nat. Commun. 2020, 11, 1731, 10.1038/s41467-020-15597-y. 32265452 PMC7138826

[19] L. Li , Z. Hu , Y. Kang , S. Cao , L. Xu , L. Yu , L. Zhang , J. C. Yu , Nat. Commun. 2023, 14, 1890, 10.1038/s41467-023-37007-9. 37019917 PMC10076521

[20] L. Li , Z. Hu , J. C. Yu , Angew. Chem. Int. Ed. 2020, 59, 20538. 10.1002/anie.202008031 32700466

[21] X. Wang , X. Zhang , Y. Wang , H. Liu , J. Wang , J. Qiu , H. L. Ho , W. Han , K. L. Yeung , Chem. Eng. J. 2011, 175, 408–416, 10.1016/j.cej.2011.08.077.

[22] Y. Zuo , X. Wang , X. Guo , Ind. Eng. Chem. Res. 2011, 50, 8485–8491, 10.1021/ie200281v.

[23] H.‐W. Shim , I.‐S. Cho , K. S. Hong , A.‐H. Lim , D.‐W. Kim , J. Phys. Chem. C 2011, 115, 16228.

[24] L. N. Demyanets , V. V. Iliukhin , A. V. Chichagov , N. V. Belov , Inorg. Mater. 1967, 3, 1938–1949.

[25] L. Li , R. P. Antony , C. S. Santos , N. Limani , S. Dieckhöfer , W. Schuhmann , Angew. Chem. Int. Ed. 2024, 63, e202406543. 10.1002/anie.202406543 38923335

[26] S. Mavrikis , M. Göltz , S. Rosiwal , L. Wang , C. Ponce de León , ChemSusChem. 2022, 15, e202102137, 10.1002/cssc.202102137. 34935302

[27] Z. Liu , W. Zhao , F. Xiao , W. Wei , Y. Sun , Catal. Commun 2010, 11, 675–678, 10.1016/j.catcom.2010.01.004.

[28] G. Y. Nigussie , Y.‐F. Tsai , T.‐C. Yang , C.‐M. Yang , S. S.‐F. Yu , J. Mater. Chem. A 2025, 13, 5261.

[29] M. Feng , Z.‐H. Luo , S. Yi , H. Lu , C. Lu , C.‐Y. Li , J.‐L. Zhao , G.‐P. Cao , Ind. Eng. Chem. Res. 2018, 57, 16227–16238, 10.1021/acs.iecr.8b03810.

[30] Y. Bai , R. Zhang , X. Ye , Z. Zhu , H. Xie , B. Shen , D. Cai , B. Liu , C. Zhang , Z. Jia , S. Zhang , X. Li , F. Wei , Nat. Nanotechnol. 2018, 13, 589–595, 10.1038/s41565-018-0141-z. 29760522

[31] Z. Zhang , R. Fang , X.‐X. Kong , M. Feng , G.‐P. Cao , H. Lu , S. Ji , P. Gao , J.‐H. Zhang , Ind. Eng. Chem. Res. 2019, 58, 19033.

[32] Y. Wu , Q. Liu , X. Su , Z. Mi , Front. Chem. China 2008, 3, 112–117, 10.1007/s11458-008-0007-2.

[33] Q. Zhang , L. Li , Q. Zhou , H. Zhang , H. Zhang , B. An , H. Ning , T. Xing , M. Wang , M. Wu , W. Wu , ACS Catal. 2023, 13, 13101–13110, 10.1021/acscatal.3c02904.

[34] J. Lu , X. Zhang , J. J. Bravo‐Suárez , T. Fujitani , S. T. Oyama , Catal. Today 2009, 147, 186.

[35] B. Taylor , J. Lauterbach , W. N. Delgass , Appl. Catal. A‐Gen. 2005, 291, 188–198, 10.1016/j.apcata.2005.02.039.

[36] Z. Song , X. Feng , N. Sheng , D. Lin , Y. Li , Y. Liu , X. Chen , X. Zhou , de Chen, C. Y. , Catal. Today 2020, 347, 102.

[37] S. Mavrikis , S. C. Perry , P. K. Leung , L. Wang , C. Ponce de León , ACS Sustainable Chem. Eng. 2021, 9, 76–91, 10.1021/acssuschemeng.0c07263.

[38] H. Bemana , N. Kornienko , iScience 2024, 27, 109482. 38558937 10.1016/j.isci.2024.109482 PMC10981096

[39] Y. Yuan , L. Chen , Z. Wan , K. Shi , X. Teng , H. Xu , P. Wu , J. Shi , Sci. Adv. 2024, 10, eado1755. 38787946 10.1126/sciadv.ado1755 PMC11122679

[40] M.‐H. Guan , L.‐Y. Dong , T. Wu , W.‐C. Li , G.‐P. Hao , A.‐H. Lu , Angew. Chem. Int. Ed. 2023, 62, e202302466. 10.1002/anie.202302466 36892310

[41] L. Wang , G. Xiong , J. Su , P. Li , H. Guo , J. Phys. Chem. C 2012, 116, 9122–9131, 10.1021/jp3017425.

[42] H. Yao , D. E. Richardson , J. Am. Chem. Soc. 2000, 122, 3220–3221, 10.1021/ja993935s.

