# Supplementary material for: An Electrocatalytic/Heterogeneous Catalytic Cascade for Selective Production of Propylene Oxide via Anodic H2O2 Generation
Source: Angew Chem Int Ed Engl. 2025 Dec 30;65(6):e21921. doi: 10.1002/anie.202521921 (PMC12865236; doi:10.1002/anie.202521921)
Supplement: Supplementary file 1 — Supporting Information [file ANIE-65-e21921-s001.docx]

Supporting Information

An Electrocatalytic/Heterogeneous Catalytic Cascade for Selective Production of Propylene Oxide via Anodic H_2_O_2_ Generation

Shubhadeep Chandra,^[a]^ Anirudha Shekhawat,^[a]^ Adarsh Koul,^[a]^ Ridha Zerdoumi,^[a,b]^ Lejing Li,*^[a]^ Wolfgang Schuhmann*^[a]^

[a] Dr. S. Chandra, A. Shekhawat, Dr. A. Koul, Dr. R. Zerdoumi, Dr. L. Li, Prof. Dr. W. Schuhmann
Analytical Chemistry – Center for Electrochemical Sciences (CES), Faculty of Chemistry and Biochemistry
Ruhr University Bochum, Universitätsstraße 150, D-44780 Bochum, Germany

[b] Dr. R. Zerdoumi, Chair for Materials Discovery and Interfaces, Institute for Materials, Faculty of Mechanical Engineering: Ruhr University Bochum, Universitätsstraße 150, 44801 Bochum, Germany

E-mail: lejing.li@rub.de; wolfgang.schuhmann@rub.de

**1 Experimental section**

**1.1 Chemicals and materials**

All chemicals were obtained commercially and used without further purification. Zn(NO_3_)_2_·6H_2_O (≥99.0%) and Na_2_WO_4_·2H_2_O (≥99.0%), maleic acid (≥99.0%) were purchased from Sigma-Aldrich. Titanium silicate molecule sieve (TS-1) was acquired from ACS Materials. TS-1 particles have a diameter of 200 to 300 nm with a Si/Ti molar ratio of 100.

**1.2 Preparation of ZnWO_4_ catalyst and ZnWO_4_ anode**

ZnWO_4_ samples were prepared through a hydrothermal process.^[1]^ Zn(NO_3_)_2_·6H_2_O and Na_2_WO_4_·2H_2_O were mixed in a 1:1 molar ratio, followed by the addition of 100 mL of distilled water. White precipitates appeared immediately, and the beaker was placed in an ultrasonic bath for 30 min to complete the precipitation reaction. The precipitates were then filtered, washed several times with distilled water, and transferred into stainless steel autoclaves with Teflon liners containing 30 mL of distilled water. After sealing, the autoclaves were heated in a convection oven at 180 °C for 24 h. The resulting products were collected by filtration, washed thoroughly with distilled water, and dried at 80 °C for 4 h before characterization.

ZnWO_4_ catalyst (120 mg) and polytetrafluoroethylene (40 mg) were dispersed in 20 mL of ethanol and sonicated for 20 min to form a uniform mixture. The cleaned carbon cloth (CC) was placed on a hot plate at 80 °C, and the catalyst mixture was drop-casted on it. The coated CC was then heat-treated at 350 °C for 30 min under argon flow. The ZnWO_4_ loading was approximately 10 mg cm⁻^2^.

**1.3 Fabrication of TS-1 loaded gas diffusion layer (GDL)**

**Preparation of CNTs@NF**

Nickel foams (NF) were ultrasonically treated with 3 M HCl solution for 15 min to remove surface oxides. The NF were then rinsed thoroughly with deionized water and acetone to remove organic residues, followed by drying in a vacuum oven overnight. The cleaned NF were immersed in a carbon nanotubes (CNTs) dispersion and stirred at room temperature (25 °C) overnight. After the reaction, a uniform black CNT film formed on the NF surface. Then the carbon nanotube decorated NF (CNT@NF) were rinsed with deionized water and dried for subsequent use.

**Loading of TS-1 onto CNTs@NF**

TS-1 powders were dispersed in deionized water under magnetic stirring. The CNTs@NF were immersed in the TS-1 suspension and stirred for 12 h to facilitate deposition. TS-1 modified CNTs@NF were then removed and dried at 383 K for 12 h. After that, TS-1 nanoparticles are evenly dispersed across the CNTs@NF surface. After TS-1 modification, CNTs are no longer visible, as they are entirely covered by the TS-1 particles. The gas diffusion layer (TS-1@GDL) was prepared by integrating TS-1 on CNTs@NF with carbon paper.

**1.4 Catalyst characterization**

X-ray diffraction (XRD) data were obtained using a Bruker D8 Discover X-ray diffractometer equipped with a Cu Kα radiation source (λ = 1.5418 Å) in the range of 5° to 70°. A small powder sample was placed on a low background silicon wafer in a PMMA holder. Field-emission scanning electron micrographs (FE-SEM) were recorded using a Quanta 3D ESEM. Transmission electron microscopy (TEM) and high-resolution TEM were obtained using a JEOL microscope (JEM-2800) with a Schottky-type emission source working at 200 kV. The powder sample was dispersed in isopropanol and 10 µL were drop-casted on a carbon-supported gold TEM grid. Prior to inductively coupled plasma mass spectrometry (ICP-MS) measurements, a perchlorate precipitate was employed to significantly reduce the potassium content since high concentrations of alkali metal ions can suppress the detection sensitivity for other elements in ICP-MS analysis. Specifically, 1 mL of sample was mixed with 0.5 mL of 70% HClO_4_ and 0.3 mL of HNO_3_. The mixture was diluted to a final volume of 10 mL with 8.2 mL ultrapure water (resistivity: 0.055 μS cm^-1^), resulting in a 10% (v/v) solution. The above solution was cooled at 4 ^o^C for 90 min to facilitate KClO_4_ precipitation, followed by filtration using 0.2 μm syringe filters. Then the liquid sample was diluted to 0.01%, 0.1%, 1% and 10% with 2% nitric acid. ICP-MS determination was performed on an iCAP-RQ (Thermo Fisher), using argon plasma in collision cell mode (KED mode) with helium as collision gas. The concentration of Zn in the anolyte after the stability test was determined to be about 201 ppb (close to the detection limit). The near-surface composition of the as-prepared ZnWO_4_ and post-electrolysis samples were investigated using X-ray photoelectron spectroscopy (XPS). An AXIS Nova spectrometer (Kratos Analytical) equipped with a monochromatic Al Kα X-ray source (1487 eV, 15 mA emission current) was used for the investigation. The pressure in the sample analysis chamber was around 10^-8^ Torr. Photoelectrons were collected in the fixed transmission mode while applying charge neutralization using an electron flood gun. A pass energy of 20 eV was used to acquire narrow spectra of the W 4*f*, Zn 3*p*, Zn 2*p*, C 1*s*, and O 1*s* regions. The binding energies of the core-level spectra were calibrated based on the C 1*s* signal at 284.8 eV. Data processing and peak fitting of different components in the XPS spectra were conducted using the ESCApe software package (Kratos). Peak fitting was performed with a combination of Gaussian and Lorentzian line shapes, and the Tougaard algorithm was used for background subtraction. ICP-MS determination was performed on an iCAP-RQ (Thermo Fisher), using argon plasma in collision cell mode (KED mode) with helium as collision gas. Nuclear magnetic resonance (NMR) spectroscopy was recorded on a Bruker 400 MHz NMR spectro­meter. The UV-vis absorption spectra were recorded on a Cary Series UV-Vis spectrometer (Agilent Technologies).

Raman spectroscopy was performed using a Lab-RAM HR Raman microscopy system (Horiba Jobin Yvon, HR550) equipped with a 532 nm laser as the excitation source, a water immersion objective (Olympus LUMFL, 60×, numerical aperture 1.0), a monochromator (1200 grooves mm^-1^ grating), and a Synapse CCD detector. Each spectrum is an average of two to five continuously acquired spectra with a collection time of 50 s each.

**1.5 Electrochemical measurements**

Electrochemical measurements were performed using a Gamry Reference 600 potentiostat/gal­vanostat in a three-electrode flow-through electrolyzer. A ZnWO_4_ anode was used as the working electrode (WE), while NF served as counter electrode (CE). The WE and CE compart­ments were separated by an anion exchange membrane (FAA-PK-130, Fumasep). A Perimax 12 peristaltic pump was employed to circulate the electrolytes through the electrolyzer. A double-junction Ag/AgCl/3 M KCl reference electrode with electrolyte solution in the outer compartment was used to protect the integrity of the reference electrode, and its potential was regularly checked against a commercial reference electrode. The RE was positioned at a fixed distance from the WE surface throughout the measurements. The H_2_O_2_ generation performan­ces of the ZnWO_4_ anode were evaluated under potentiostatic conditions. Electrochemical impedance spectroscopy was conducted with an AC amplitude of 10 mV_pp_ over a frequency range of 1.0 to 100 kHz to determine the uncompensated solution resistance R_u_. After iR-drop compensation, the substrate potential was recalculated relative to the reversible hydrogen electrode (RHE) according to E_RHE_ = E_Ag/AgCl/3M KCl_ + 0.21 + 0.059 × pH – *i* × R_u_, and the pH values of the electrolytes were measured using a pH meter (FE28, Mettler Toledo). The elec­trode area of the ZnWO_4_ anode was maintained at 1 cm^2^ throughout all the experiments. The catalytic performance for propylene oxide production was subsequently assessed using 5 mL of the same electrolyte under continuous propylene feeding.

**1.6 Reaction Workup and Quantification of Liquid Products**

The H_2_O_2_ concentration was quantified by spectrophotometric determination of I_3_^-^.^[2]^ Prior to quantification, the pH of the aliquots was adjusted to 7 by adding sulfuric acid. The amount of propylene oxide and propylene glycol formed during the reactions was quantified by ^1^H-NMR spectroscopy using maleic acid as an internal standard. Samples were prepared by combining 200 μL of electrolyte with 200 μL of H_2_O and 100 μL of a 10 mM maleic acid solution in D_2_O. The ^1^H probe was tuned and locked on the D_2_O solvent, with gradient shimming and auto-gain applied. Water peak suppression was performed, and 16 scans were collected per sample with a 6 s relaxation delay.

**1.7 Electron efficiency calculations** **of the tandem anodic H_2_O_2_ generation and propylene epoxidation**

The Faradaic efficiency (FE) of H_2_O_2_ was calculated using the following formula:

$\mathrm{FE}_{H2O2}\left( \% \right)=\frac{n \times z \times F}{Q}$ × 100 %

where n is the moles of H_2_O_2_ formed, z is the number of electrons required to form H_2_O_2_, F is the Faraday constant (96485 C mol^–1^), and Q is the total charge passed.

The propylene oxide (PO) selectivity was calculated using the formula:

$PO Selectivity \left( \% \right)=\frac{n_{PO}}{n_{PO} + n_{PG}}$ × 100 %

Where $n_{PO}$ and $n_{PG}$ indicate the number of moles of PO and propylene glycol (PG) formed, respectively. The overall efficiency of the tandem system depends on the efficiency of each step, namely, anodic H_2_O_2_ generation and the utilization of H_2_O_2_ for propylene epoxidation. We define the concept of H_2_O_2_ utilization efficiency (UE_H2O2_) as the fraction of total generated H_2_O_2_ that contributes to the formation of PO and PG.

H_2_O_2_ utilization efficiency (UE_H2O2_) $(\%)$ = $\frac{n_{PO} + n_{PG}}{n_{H2O2 -}{nr}_{H2O2}}$ ${\times100\%}$

Where $n_{PO}+n_{PG}$ represents the total amount of PO and PG formed, $n_{H2O2}$ represents the total amount of H_2_O_2_ produced by the ZnWO_4_ anode and ${nr}_{H2O2}$ represents the remaining H_2_O_2_ after the respective time interval.

Then the electron efficiency (EE) for PO and PG production can be expressed as:

EE = $\mathrm{FE}_{H2O2}{\times\mathrm{UE}}_{H2O2}$

**2 Supplementary Figures**


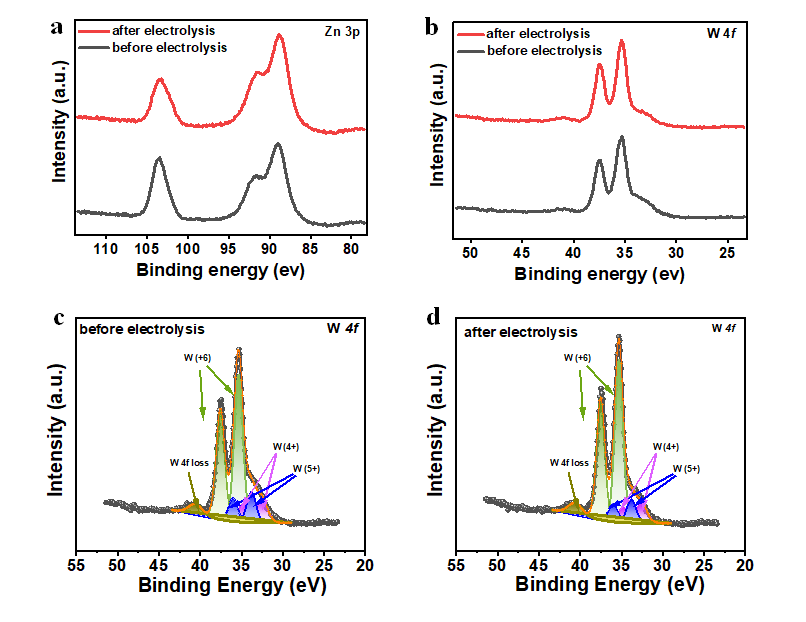


**Figure S1.** (a) High-resolution XPS spectra of the Zn 3*p* region before and after electrolysis. (b-d) High-resolution XPS spectra of the W 4*f* region before and after electrolysis. Electrolysis was performed at 2.4 V vs. RHE on the ZnWO_4_ anode in 2 M KHCO_3_/K_2_CO_3_ (pH 9) electrolyte containing 10 % acetonitrile (ACN).


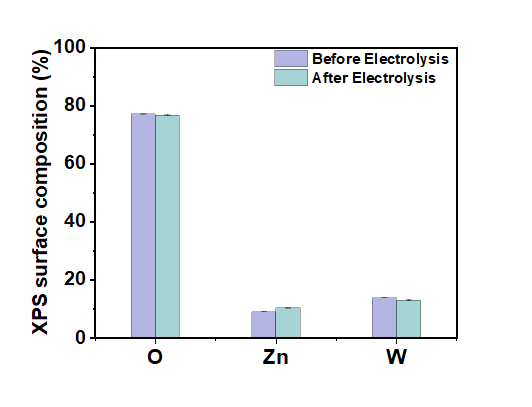


**Figure S2.** Near-surface composition of O, Zn, and W determined using XPS on the samples before and after electrolysis. Electrolysis was performed at 2.4 V vs. RHE on the ZnWO_4_ anode in 2 M KHCO_3_/K_2_CO_3_ (pH 9) electrolyte containing 10 % ACN. Error bars represent the standard deviation from two independent experiments.


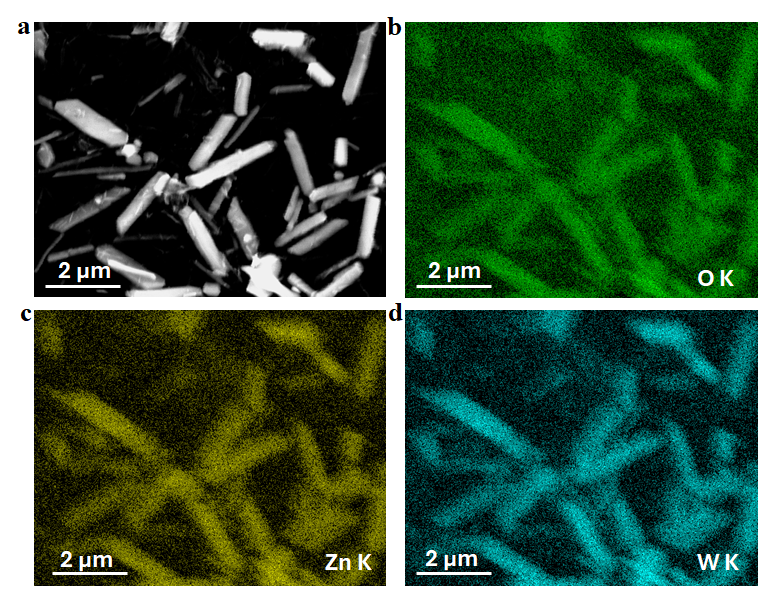


**Figure S3.** (a) SEM images of ZnWO_4_ anode before electrolysis (b) Corresponding elemental mapping of ZnWO_4_ anode.


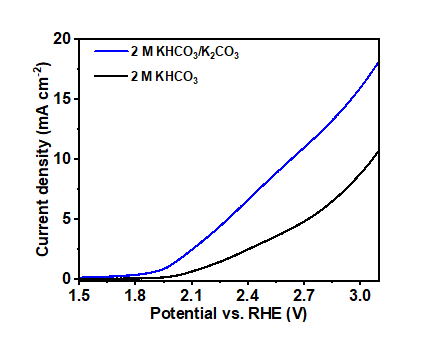


**Figure S4.** LSV curves recorded in 2 M KHCO_3_ and a mixture of 2 M KHCO_3_/K_2_CO_3_ (pH 9) electrolyte. The scan rate was 50 mV s^−1^.


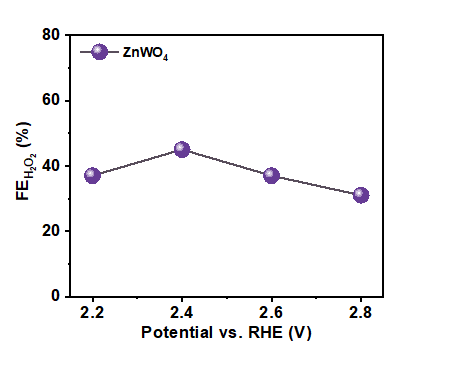


**Figure S5.** FE_H2O2_ of the ZnWO_4_ anode at different potentials in 2 M KHCO_3_.


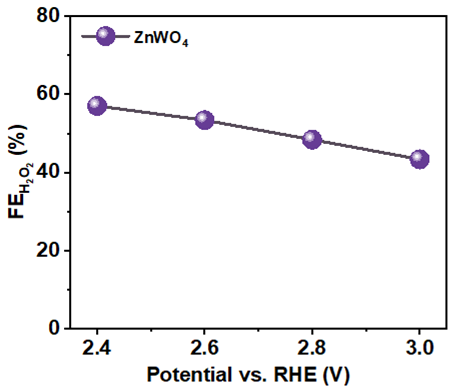


**Figure S6.** FE_H2O2_ of the ZnWO_4_ anode at different potentials in 2 M K_2_CO_3_.


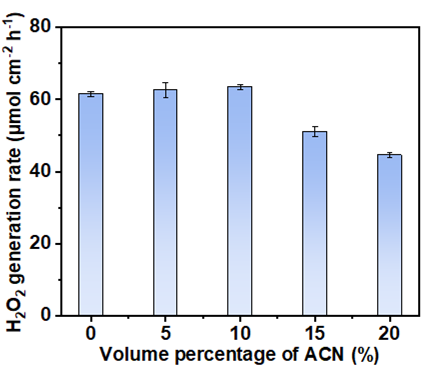


**Figure S7.** H_2_O_2_ generation rate on ZnWO_4_ anode at 2.4 V vs. RHE in 2 M KHCO_3_/K_2_CO_3_ (pH 9) electrolyte mixed with different volume percentages of ACN. Error bars represent the standard deviation from three independent experiments.


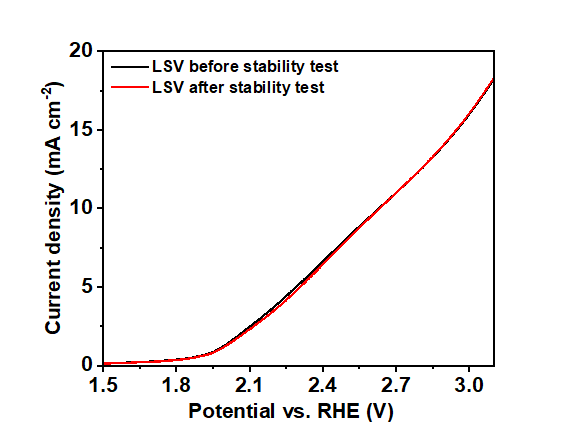


**Figure S8.** LSV curves recorded before and after a stability test in 2 M KHCO_3_/K_2_CO_3_ (pH 9) electrolyte containing 10 % ACN. The scan rate was 50 mV s^−1^.


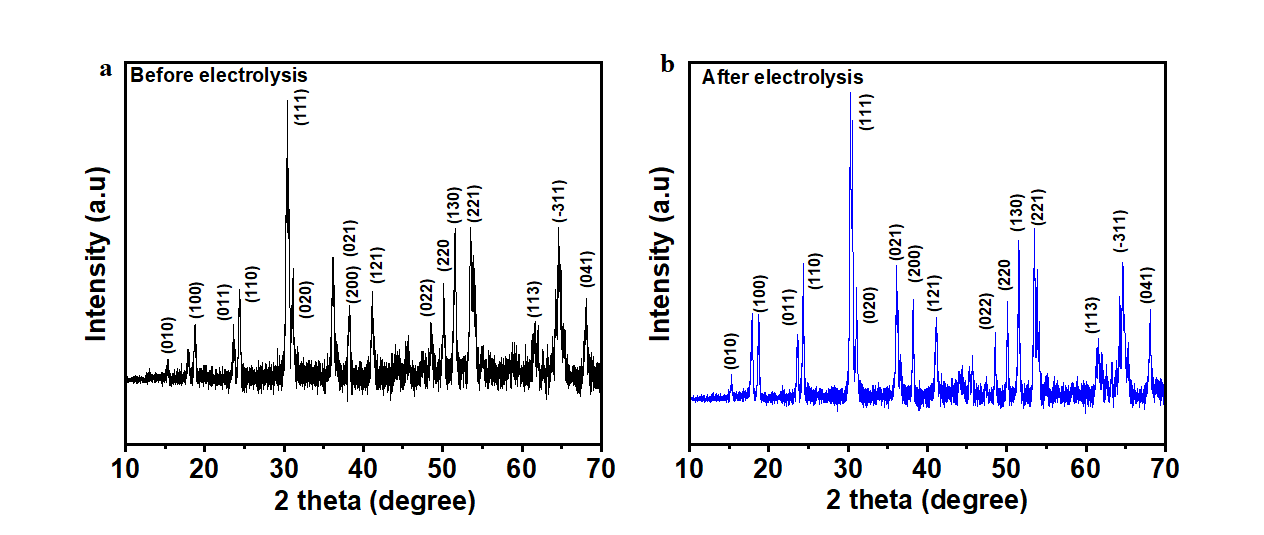


**Figure S9.** XRD patterns of ZnWO_4_ anode before and after electrolysis. Electrolysis was performed at 2.4 V vs. RHE on ZnWO_4_ anode in 2 M KHCO_3_/K_2_CO_3_ (pH 9) electrolyte containing 10 % ACN.


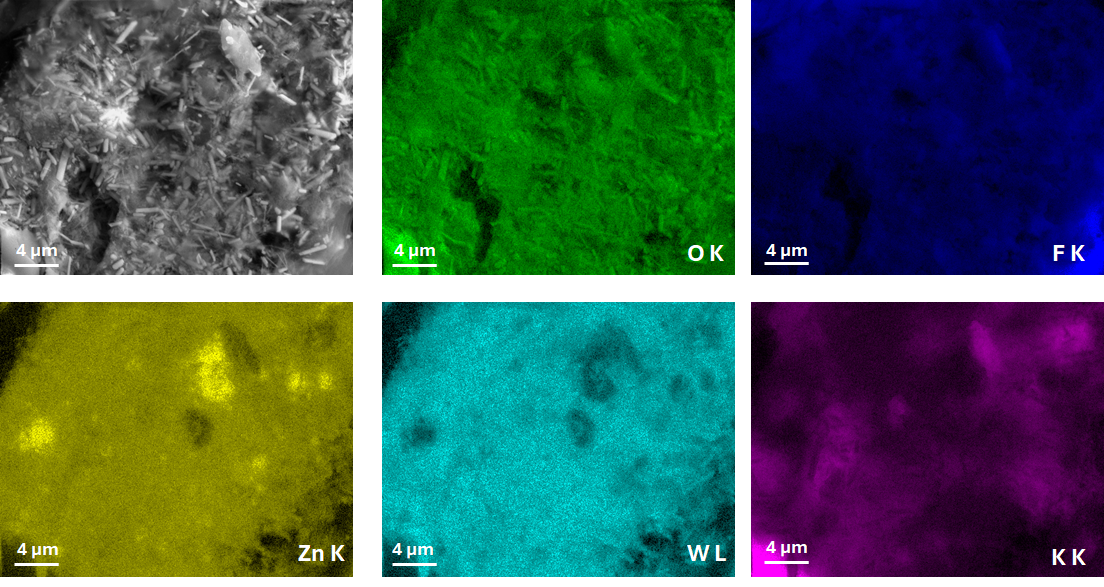


**Figure S10.** SEM images of ZnWO_4_ anode after electrolysis and its corresponding elemental mapping. Electrolysis was performed at 2.4 V vs. RHE on the ZnWO_4_ anode in 2 M KHCO_3_ /K_2_CO_3_ (pH 9) electrolyte containing 10 % ACN.


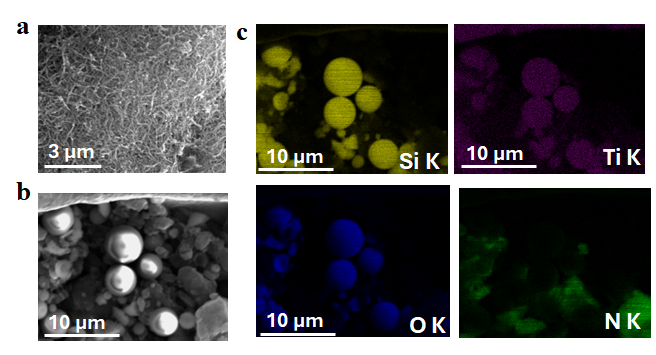


**Figure S11.** (a) SEM images of carbon-nanotubes (CNTs) decorated nickel foam (CNT@NF). (b) SEM image of TS-1 immobilized CNT@NF serving as gas diffusion layer (GDL). (c) The corresponding elemental mapping of the TS-1@GDL. The SEM image reveals nickel foam uniformly covered with randomly oriented CNTs. The dense and uneven distribution of CNTs indicates the presence of aggregated and confined pores, resulting in a coarse surface morphology.


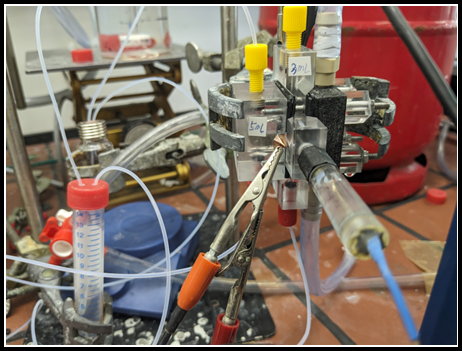


**Figure S12.** The self-designed flow-through electrolyzer used for propylene epoxidation. The ZnWO_4_ anode was positioned in close proximity to the TS-1@GDL. Additionally, employing a compact flow-through cell with a small electrolyte volume (?mL) further promotes localized H_2_O_2_ enrichment near TS-1@GDL, boosting epoxidation efficiency.


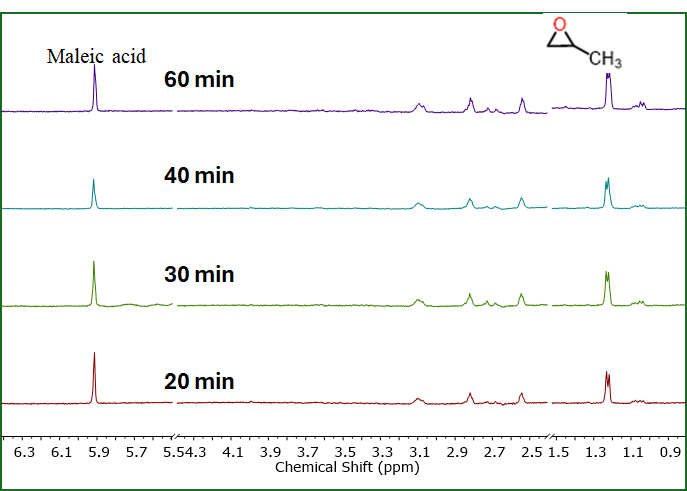


**Figure S13.** ^1^H-NMR spectra of products measured after different time intervals**.** Electrolysis was performed at 2.4 V vs. RHE on the ZnWO_4_ anode in 2 M KHCO_3_/K_2_CO_3_ (pH 9) electrolyte containing 10 % ACN.


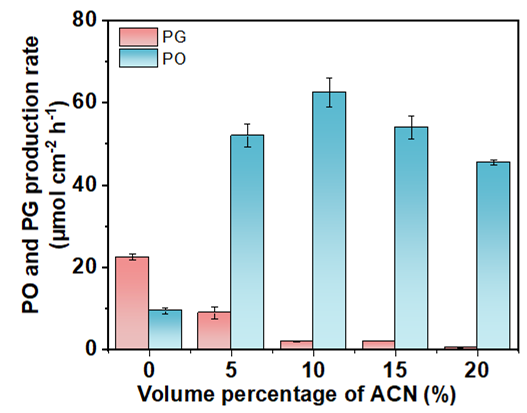


**Figure S14.** PO and PG production rate in 2 M KHCO_3_/K_2_CO_3_ (pH 9) electrolyte mixed with different volume percentages of ACN**.** Error bars represent the standard deviation from three independent experiments.


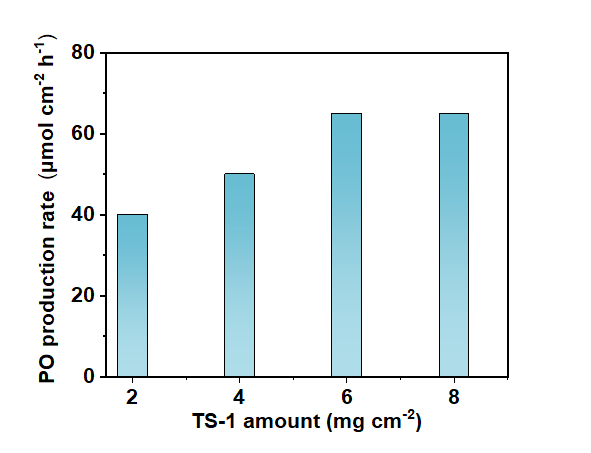


**Figure S15.** PO production rate at 2.4 V vs. RHE on ZnWO_4_ anode using different amounts of immobilized TS-1 on GDL for propylene epoxidation in 2 M KHCO_3_/K_2_CO_3_ (pH 9) electrolyte containing 10 % ACN**.**


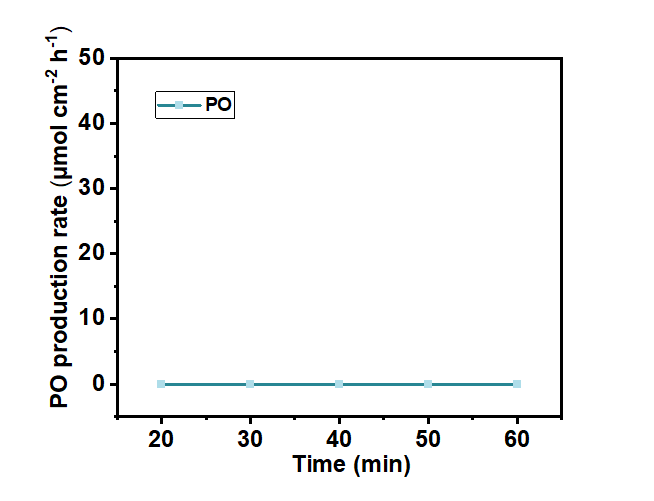


**Figure S16.** PO production rate at 2.4 V vs. RHE on the ZnWO_4_ anode in 2 M KHCO_3_/K_2_CO_3_ (pH 9) electrolyte containing 10 % ACN, in the absence of TS-1.


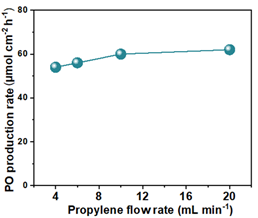


**Figure S17.** Production rate of PO at different propylene flow rates. Electrolysis was performed at 2.4 V vs. RHE on the ZnWO_4_ anode in 2 M KHCO_3_/K_2_CO_3_ (pH 9) electrolyte containing 10 % ACN.


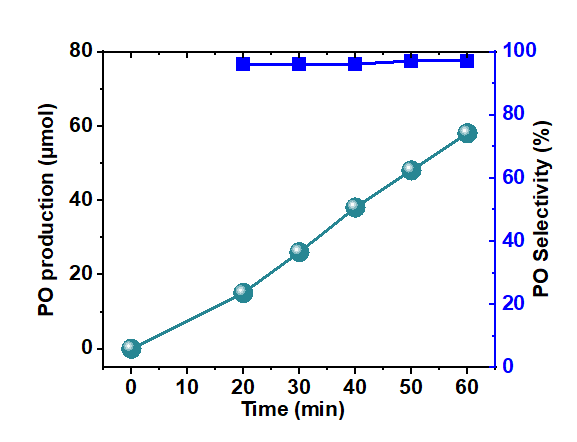


**Figure S18.** Production and selectivity of PO over time at 2.4 V vs. RHE on the ZnWO_4_ anode in 2 M KHCO_3_/K_2_CO_3_ (pH 9) electrolyte containing 10 % ACN.


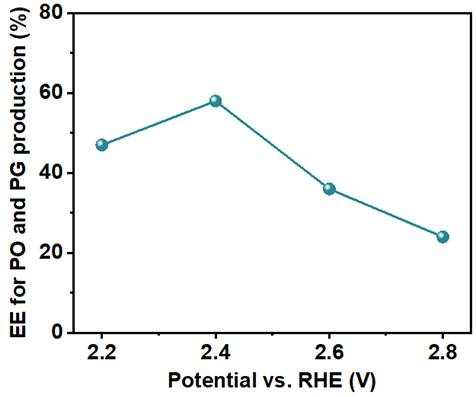


**Figure S19.** Electron efficiency (EE) for PO and PG production at different potentials. Electrolysis was performed at different potentials in a 2 M KHCO_3_/K_2_CO_3_ (pH 9) electrolyte containing 10 % ACN.


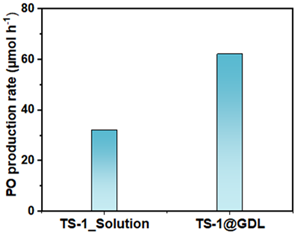


**Figure S20.** Production rate of PO using the same amount of TS-1 suspended in electrolyte solution (TS-1_Solution) and immobilizing on the GDL surface (TS-1@GDL). Electrolysis was performed at 2.4 V vs. RHE on the ZnWO_4_ anode with the anode area kept the same to ensure comparable H₂O₂ production, in 2 M KHCO_3_/K_2_CO_3_ (pH 9) electrolyte containing 10 % ACN.


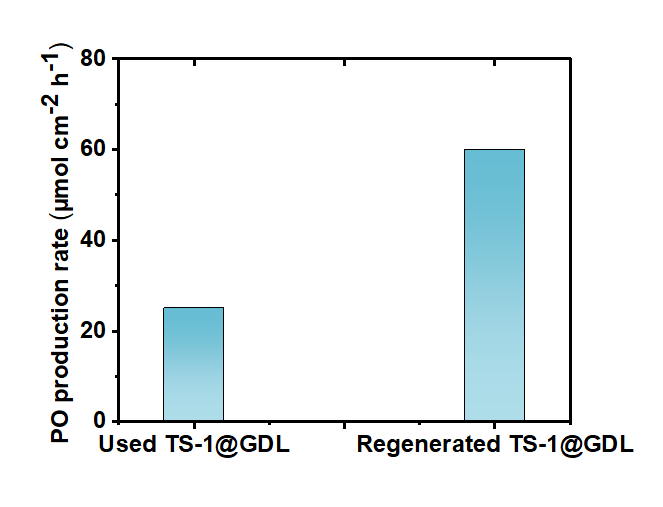


**Figure S21.** Production rate of PO over used and regenerated TS-1@GDL under optimized conditions. Electrolysis was performed at 2.4 V vs. RHE on the ZnWO_4_ anode in 2 M KHCO_3_/K_2_CO_3_ (pH 9) electrolyte containing 10 % ACN.

**Figure S22.** (a) SEM images of TS-1@GDL before electrolysis. (b) SEM image of TS-1@GDL after electrolysis (TS-1@GDL post-treated by calcination). Furthermore, no distinct morphological difference can be found in SEM compared to the fresh TS-1@GDL. This indicates that the TS-1 catalysts remained well anchored to the GDL with minimal detachment.

***In situ* Fourier Transform Infrared (FTIR) measurements** were conducted using a three-electrode setup. ZnWO_4_ catalyst was integrated into a borehole electrode, serving as the working electrode. A platinum mesh was employed as the counter electrode, and Ag/AgCl/3 M KCl electrode served as the reference electrode. The distance between the electrode and the internal reflection element (IRE) was precisely set at 20 µm. Electrolysis was performed in 2 M KHCO_3_/K_2_CO_3_ (pH 9) electrolyte. Spectra were acquired using a Bruker Tensor 27 spectrometer with a commercial A530/P reflection unit. Each spectrum was recorded by accumulating 200 scans over 30 s.


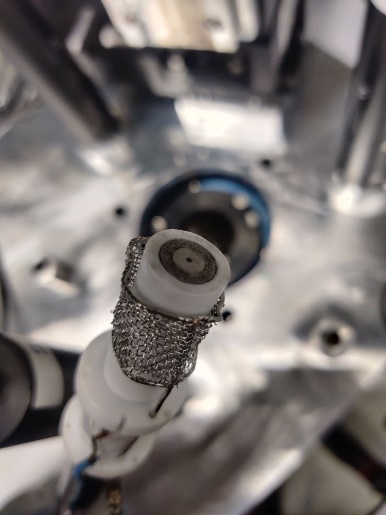


**Figure S23.** Electrode used for collecting FTIR spectra.


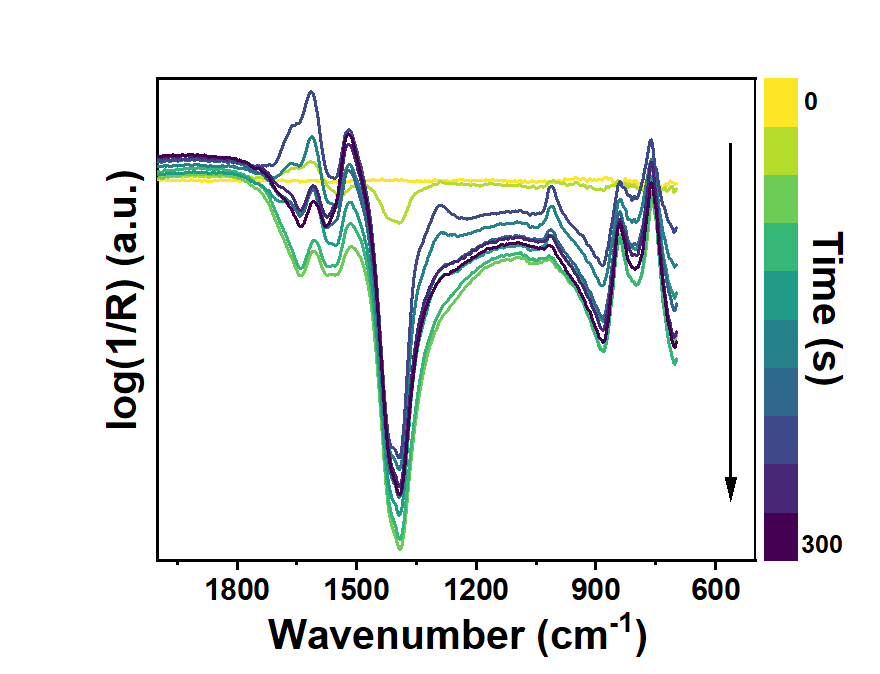


**Figure S24.** IR spectra measured as a function of applied potential, ranging from 2.0 to 3.0 V vs. RHE, showing the formation of percarbonate (HCO_4_^-^).


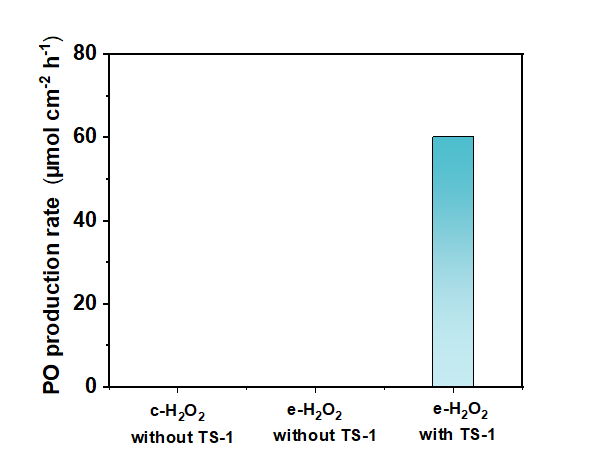


**Figure S25.** Production rate of PO using commercial H_2_O_2_ without TS-1 in KHCO_3_ solution using *in situ* generated H_2_O_2_ with and without TS-1@GDL. Electrolysis was performed at 2.4 V vs. RHE on the ZnWO_4_ anode in 2 M KHCO_3_/K_2_CO_3_ (pH 9) electrolyte containing 10 % ACN. c-H_2_O_2_ means commercial H_2_O_2_ in KHCO_3_. e-H_2_O_2_ means electrochemically produced H_2_O_2_ in 2 M KHCO_3_/K_2_CO_3_ (pH 9) electrolyte containing 10 % ACN.

**Table S1 Reported PO production rates with TS-1.**

| Catalysts | Reactants | Reaction Temperature (°C) | PO (μmol g_cat_^-1^ h^-1^) | PO selectivity (%) | Ref |
| --- | --- | --- | --- | --- | --- |
| 0.03 Au/TS-1 | C_3_H_6_ + H_2_ + O_2_ | 140 | 810 | 89 | ^[3]^ |
| 0.02 Au/TS-1 | C_3_H_6_ + H_2_ + O_2_ | 200 | 660 | - | ^[4]^ |
| Au/HTS-1 | C_3_H_6_ + H_2_ + O_2_ | 200 | 1500 | - | ^[5]^ |
| CN-Ar/TS-1 | C_3_H_6_ + O_2_ | RT | 5515 | 99.1 | ^[6]^ |
| CoPi/BiVO_4_/TS-1 | C_3_H_6_ + O_2_ | RT | 269 | 98 | ^[7]^ |
| ZnWO_4_/ TS-1 | C_3_H_6_ + H_2_O | RT | 6200 | 97 | **This work** |

In this tandem reaction system, the performance of electrochemically generated H_2_O_2_ determined the rate of PO production. Therefore, the production rate of PO is calculated based on the amount of ZnWO_4_ used per cm^2^ of anode.

**Table S2 Comparison of performance with other photocatalytic or electrocatalytic tandem systems that utilize in situ generated H₂O₂ for propylene epoxidation.**

| Catalysts | Conditions | H_2_O_2_  Productivity | H_2_O_2_ conversion | PO productivity | PO selectivity | Ref |
| --- | --- | --- | --- | --- | --- | --- |
| CN-Ar/TS-1 | 10 W blue LED lamp (420-430nm) | 5.77 μmol mg^-1^ h^-1^ | 86.31 % | 5.52 μmol mg^-1^ h^-1^ | 99.1 % | ^[6]^ |
| TiO_2_,  Co-N/CNT, TS-1 | 300W Xe lamp | 3.78 μmol cm^-2^ h^-1^ | 84.12 % | 3.09 μmol cm^-2^ h^-1^ | 97.5 % | ^[7]^ |
| BiVO_4_,  Co-N/CNT, TS-1 | 420 nm cut-off  filter | 4.51 μmol cm^-2^ h^-1^ | 81.08 % | 3.50 μmol cm^-2^ h^-1^ | 99.1 % |  |
| O-doped  C black /TS-1 | -0.75 V vs. RHE | ^a^14.8 μmol mg^-1^ h^-1^ | 47.60 % | 4.28 μmol mg^-1^ h^-1^ | 79.7 % | ^[8]^ |
| SnO_2_/BiVO_4_, hydrogel TS-1 | 300W Xe lamp,  1.2 V vs. RHE | 57.58 μmol cm^-2^ h^-1^ | 94.06 % | 33.04 μmol cm^-2^ h^-1^ | 91.05 % | ^[9]^ |
| ZnWO_4_/ TS-1 | 2.4 V vs. RHE | 64 μmol cm^-2^ h^-1^ | 98 % | 62 μmol cm^-2^ h^-1^ | 97 % | **This work** |

^a^The H_2_O_2_ production rate recalculated by the standard of per gram of catalyst according to the mass ratio of O-doped C black/TS-1.

**References**

[1] H.-W. Shim, I.-S. Cho, K. S. Hong, A.-H. Lim, D.-W. Kim, “Wolframite-type ZnWO_4_ Nanorods as New Anodes for Li-Ion Batteries” *J. Phys. Chem. C* **2011**, *115*, 16228.

[2] N. V. Klassen, D. Marchington, H. C. McGowan, “H_2_O_2_ Determination by the I_3_^-^ Method and by KMnO_4_ Titration” *Anal. Chem.* **1994**, *66*, 2921.

[3] B. Taylor, J. Lauterbach, W. N. Delgass, “Gas-phase epoxidation of propylene over small gold ensembles on TS-1” *Appl. Catal. A-Gen.* **2005**, *291*, 188.

[4] J. Lu, X. Zhang, J. J. Bravo-Suárez, T. Fujitani, S. T. Oyama, “Effect of composition and promoters in Au/TS-1 catalysts for direct propylene epoxidation using H_2_ and O_2_” *Catal. Today* **2009**, *147*, 186.

[5] Z. Song, X. Feng, N. Sheng, D. Lin, Y. Li, Y. Liu, X. Chen, X. Zhou, de Chen, C. Yang, “Propene epoxidation with H_2_ and O_2_ on Au/TS-1 catalyst: Cost-effective synthesis of small-sized mesoporous TS-1 and its unique performance” *Catal. Today* **2020**, *347*, 102.

[6] Q. Zhang, L. Li, Q. Zhou, H. Zhang, H. Zhang, B. An, H. Ning, T. Xing, M. Wang, M. Wu, W. Wu, “Boosting C_3_H_6_ Epoxidation via Tandem Photocatalytic H_2_O_2_ Production over Nitrogen-Vacancy Carbon Nitride” *ACS Catal.* **2023**, *13*, 13101.

[7] M. Ko, Y. Kim, J. Woo, B. Lee, R. Mehrotra, P. Sharma, J. Kim, S. W. Hwang, H. Y. Jeong, H. Lim, S. H. Joo, J.-W. Jang, J. H. Kwak, “Direct propylene epoxidation with oxygen using a photo-electro-heterogeneous catalytic system” *Nat. Catal.* **2022**, *5*, 37.

[8] X.-C. Liu, W.-K. Yao, B.-Y. Su, Y.-H. Hong, T. Wang, Z.-Y. Zhou, S.-G. Sun, “Efficient Conversion of Propylene to Propylene Glycol by Coupling H₂O₂ Electrosynthesis and TS-1 Thermocatalysis” *Electrochem. Commun.* **2023**, *151*, 107510.

[9] **Y. An, X. Yang, R. Wang, Y. Gu, Y. Min, T. Zhang, J. Shen, K. Zhang,** “Improving Tandem Epoxidation Efficiency via Hydrogel Confinement Effect toward Photoelectrochemical Propylene Oxide Synthesis” Angew. Chem. Int. Ed. **2025**, e202518020.
